# Bisphosphonates adherence for treatment of osteoporosis

**DOI:** 10.1186/1755-7682-6-24

**Published:** 2013-05-24

**Authors:** Helena Parente Vieira, Ingrid Almeida Leite, Thayga Maria Araújo Sampaio, Juliane dos Anjos de Paula, Ankilma do Nascimento Andrade, Luiz Carlos de Abreu, Vitor E Valenti, Flavia C Goulart, Fernando Adami

**Affiliations:** 1 Laboratory of Studies Design and Scientific Writing, Faculty of Medicine ABC, Av. Príncipe de Gales, 821, Santo André, SP, 09060-650, Brazil; 2 Estacio Faculty of Medicine, Avenida Tenente Raimundo Rocha, 515, Juazeiro do Norte, Ceara, 63040-360, Brazil; 3 Department of Speech Language and Hearing Therapy, Faculty of Philosophy and Sciences, UNESP, Av. Hygino Muzzi Filho, 737, Marilia, SP, 17525.900, Brazil

**Keywords:** Bone and bones, Bisphosphonates, Medication adherence, Osteoporosis

## Abstract

**Background:**

Osteoporosis is a disease of bone metabolism in which bisphosphonates (BPS) are the most common medications used in its treatment, whose main objective is to reduce the risk of fractures. The aim of this study was to conduct a systematic review on BPs adherence for treatment of osteoporosis.

**Methods:**

Systematic review of articles on BPs adherence for treatment of osteoporosis, indexed on MEDLINE (via PubMed) databases, from inception of databases until January 2013. Search terms were “Adherence, Medication” (MeSH term), “Bisphosphonates” (MeSH term), and “Osteoporosis” (MeSH term).

**Results:**

Of the 78 identified studies, 27 met the eligibility criteria. Identified studies covered a wide range of aspects regarding adherence and associated factors, adherence and fracture, adherence and BPs dosage. The studies are mostly observational, conducted with women over 45 years old, showing low rates of adherence to treatment. Several factors may influence adherence: socio-economic and cultural, participation of physicians when guidance is given to the patient, the use of bone turnover markers, and use of generic drugs. The monthly dosage is associated with greater adherence compared to weekly dosage.

**Conclusions:**

Considering the methodological differences between the studies, the results converge to show that adherence to treatment of osteoporosis with BPs is still inadequate. Further experimental studies are needed to evaluate the adherence and suggest new treatment options.

## Background

Osteoporosis is the most common disease of bone metabolism, it is characterized by a reduction in bone mineral density (BMD), with consequent increased risk of fractures of the spine, hip and other parts [1]. It mainly affects postmenopausal women and it is currently considered a public health problem, since bone fractures increases significantly the morbidity and mortality of affected patients, especially hip fracture, which increases mortality up to 20% [2].

Treatment of this disease primarily focus in preventing fractures, additionally the drugs most commonly used in clinical practice are the bisphosphonates (BPs) (alendronate, risedronate, clodronate, ibandronate, zolendronic acid), which act by inhibiting bone resorption mediated by osteoclasts [3]. These drugs reduce the incidence of vertebral fractures by 40 to 50% and non-vertebral fractures by 20 to 40% [4]. However, since it is a long-term treatment, such as in other chronic diseases (hypertension, diabetes mellitus), non-adherence to these medications are common: studies suggest that only 50% of patients continue therapy for 12 months and 43% between 13 to 24 months [5].

In describing the adherence to treatment, some terms are important and must be understood. Compliance is the way the patient follows the prescribed orientations (prescribed interval, dosage) and persistence is the starting time until discontinuation of therapy; compliance is often evaluated by measuring the medication possession ratio (MPR), defined as the ratio between the prescribed interval lof medication use and the real interval(assuming full compliance) [6]. In most studies, the optimal MPR is > = 80% [7].

The reasons for treatment noncompliance are diverse, including side effects, such as esophageal irritation, and the absence of the disease symptoms [8].

Taking into account that a systematic review is a review of a clearly formulated question that directs the search of the literature, this systematic review will address the following question: “How is BPs adherence for treatment of osteoporosis?”.

Considering the importance of this topic for public health, a systematic review of articles regarding BPs adherence for treatment of osteoporosis will be presented.

## Methods

A systematic search of published articles was conducted only in MEDLINE(via PubMed), started on June 2012 and finished on January 2013. Initially, MEDLINE database was searched using the field “MeSH Terms” and Boolean operator AND in “PubMed Advanced Search Builder” tool with the search terms:

#1 “Adherence, medication” (MeSH term);

#2 “Bisphosphonates” (MeSH term);

#3 “Osteoporosis” (MeSH term).

The following search was performed: #1 AND #2 AND #3.

The articles analysis followed previously determined eligibility criteria. Inclusion criteria wereas follows:a) manuscripts written in English; b) articles about BPs adherence for treatment of osteoporosis; c) original articles with online accessible full text; d) prospective or retrospective observational (analytical or descriptive, except case reports), experimental or quasi-experimental studies. Exclusion criteria were: a) other designs, such as case reports, case series, review of literature and commentaries; b) non-original studies, including editorials, reviews, preface, brief communication, and letters to the editor; c) studies including only men.

Subsequently, each included article was read in full, and then data were extracted and entered into a form that included authors, publication year, description of the study design and main findings. Some of the studies discuss about compliance and persistence, since they are terms to describe adherence. For each study, data were extracted independently by two authors. Discrepancies were resolved by consensus between the authors.

Finally, for heuristic reasons, articles were grouped in 3 themes:adherence and associated factors; adherence and fracture; adherence and BPs dosage.

## Results

Initially, the search strategy resulted in 78 references from MEDLINE database.

**Figure 1:**
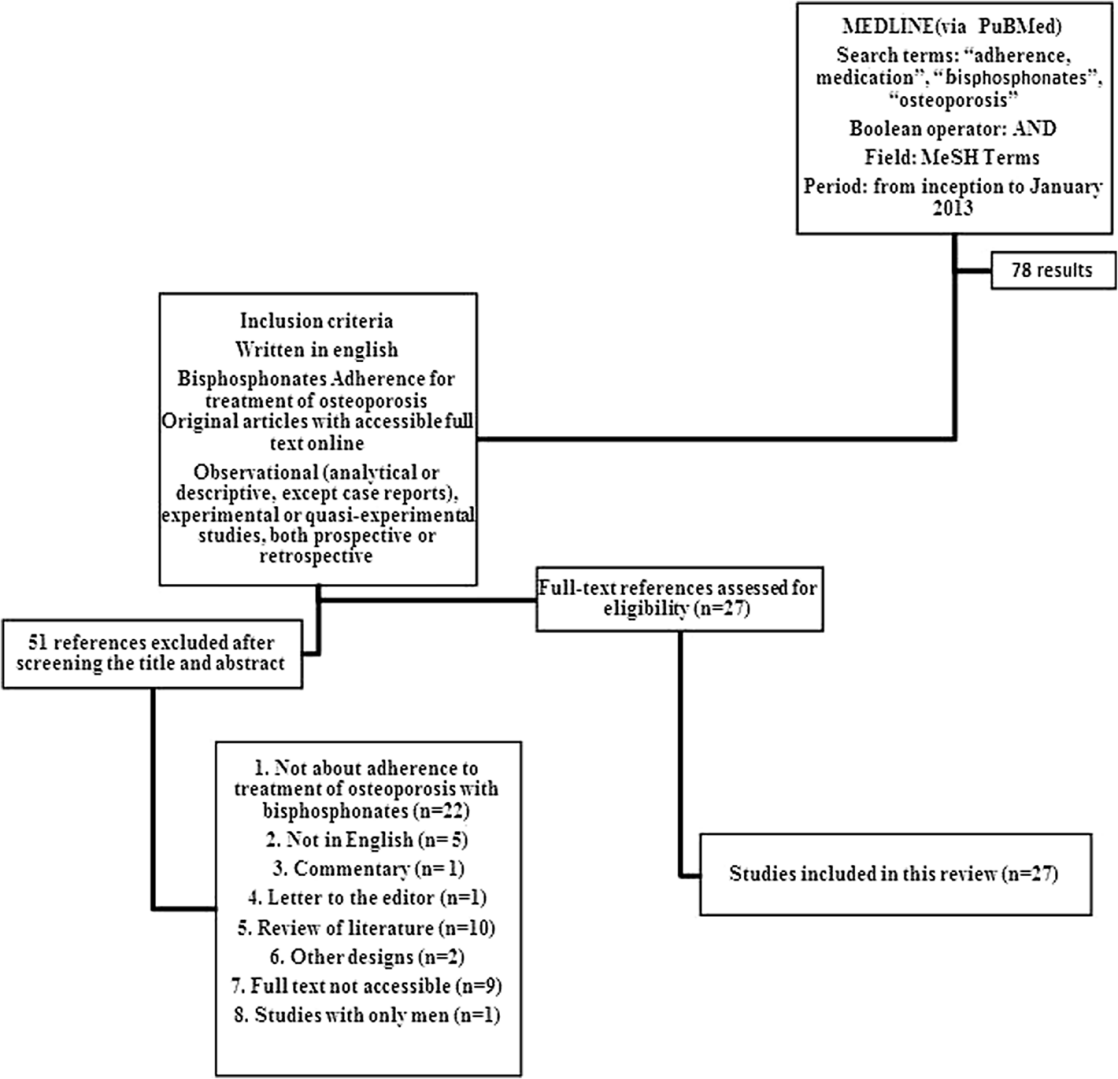
Flow chart showing study selection for the review: search strategy, number of records identified, includedand excluded, and the reasons for exclusions.

From this total, after screening the title and abstract of the identified studies for eligibility based on study inclusion criteria, 51 (71,83%) were excluded and 27(28,17%) articles were separated and included in the final sample (Figure 1).

Table 1 provides an overview of all studies included in the final sample and characteristics of studies used during the data analysis process. Study designs included 7 experimental studies and 20 observational studies [4-23]. The 27 studies were distributed in 3 themes, previously determined as follows: adherence and associated factors (20 studies) [1,2,5,7-9,11-13,15,16,18-22],[24-27], adherence and fracture (2 studies) [4,17]; adherence and dosage of BPs (5 studies) [3,6,10,14,23].

**Table 1:** Bisphosphonates adherence for treatment of osteoporosis: studies and main findings

**Author (Year)**	**Study design**	**Sample**	**Main findings**
Barret-Connor et al. [5]	Cohort study	2,405 women on osteoporosis medications- 76% taking BP	Lower treatment satisfaction was associated with 22% to 67% increased risk of discontinuation/switching osteoporosis medication during 1styear of follow-up
Ström, et al. [9]	Cohort study	36,433 participants taking risedronate or alendronate	Automatic generic substitution may have reduced persistence in participants taking alendronate. No difference was observed in persistence with proprietary risedronate during the same period.
Roux et al. [27]	Randomized controlled trial	212 women with post menopausal osteoporosis (interventions group) and 285 women with osteoporosis post menopausal in control group- multicenter study in France	This study failed to demonstrate that monitoring a serum bone turnover marker impact the persistence with monthly ibandronate treatment.
Palacios et al. [20]	Observational, prospective, multicenter trial	174 women taking weekly alendronate	Treatment with Alendronate in women with postmenopausal osteoporosis reduces the urinary excretion of the bone turnover biomarker N-telopeptide (NTx). The probability of achieving a clinically significant reduction is greater in those women with higher baseline levels of NTx and in women who comply with treatment.
Lai et al. [22]	Cross-sectional observational study	1,130 survivors of minimal trauma hip fracture admitted to a hip fracture unit (19.2% rural patients). Following fracture, only 623 patients (55.1%) were available.	Before fracture, fewer rural patients had taken BPs (7.7% versus 13.3%). Following fracture, more rural then urban patients were significantly non-compliant with BPs (44% versus 52.4%). The compliance among both rural and urban patients decreased, following hip fracture.
Bryl et al. [2]	Randomized controlled trial	42 physicians from 5 medical centers and 656 patients (Therapeutic program: Alendronate 70 mg)	56% of patients regarded the therapy as convenient. Patients more often accepted their disease and treatment if their physicians obtained high scores in the Social Competence Questionnaire. When physician competence regarding close emotional contact was high, only 15% of the patients revealed symptoms of fear of disease and treatment, in comparison to 40% of the patients, if the competence of the physician was low.
Curtis et al. [23]	Cohort study	775 taking zoledronate; 275 taking ibandronate (comparison group 1); 571 taking ibandronate (the first year that ibandronate was available- comparison group 2).	Using all available data (minimum 18 months, maximum 27 months), the proportion of patients with high adherence for the zoledronate and the 2 ibandronate cohorts was 62.8% versus 36.0% and 33.3%. But approximately 30% of patients taking zoledronate did not receive a second infusion.
Devold et al. [18]	Cohort study	7,610 patients, all incident taking alendronate.	In women, the most important factors for being adherent were advanced age and high income. In men, a middle educational level predicted adherence.
Devine et al. [10]	Cohort study	22,363 new users of an oral BP(alendronate, risedronate, or ibandronate). Weekly cohort, n = 15,228; Monthly cohort, n = 7,225.	Patients receiving oral BPs on a monthly basis showed higher rates of medication compliance compared to weekly dosage in our study. However, compliance with BPs among all new patients was suboptimal (compliance- 43%)
Burden et al. [11]	Cohort study	451,113 new BP patients: alendronate (5, 10, and 70 mg), cyclical etidronate and risedronate (5 /35 mg)	Persistence with therapy declined from 63% at 1 year to 46% at 2 years and 12% at 9 years. Most patients experienced one or more extended gaps in BP therapy.
Hadji et al. [13]	Cohort study	4,147 women treated with oral BP	Persistence rates after 1 and 2 years were 27.9% and 12.9%, respectively, and 66.3% of women were compliant. After 24 months of therapy, compliant women had fewer fractures than non-compliant women. Compliance and persistence were inadequate.
Kuzmanovaet al. [1]	Randomized Controlled Trial	341 postmenopausal women taking -weekly alendronate or mensal ibandronate	There was a very good patient medication adherence of the study subjects to the 24-month treatment with BPs. MPR ranged from 0,93 to 1,0. The patient medication persistence dropped significantly at the end of month 12.
Lai et al. [24]	Randomized controlled trial	198 patients( weekly alendronate or risedronate) : intervention = 100 (received a ‘counselling package’); control = 98 (no counselling).	When adherence was assessed by pill count, the intervention group showed a significantly higher adherence. Overall, persistence at 1 year was high and similar between groups.
Montori et al. [25]	Randomized controlled trial	100 patients: the control group received the National Osteoporosis Foundation booklet, “Boning Up On Osteoporosis: A Guide To Prevention and Treatment.”	Most patients exhibited optimal medication adherence and persistence at 6 months. Analyses of adherence or persistence did not show any significant effect of the decision aid on 6-month adherence BPs.
Ojeda- Bruno et al. [15]	Cohort study	683 patients older than 50 years with a fragility fracture were appointed for a clinical visit	Attendance of scheduled visits was associated with adherence to BPs.
Cheen et al.[12]	Retrospective observational study	798 patients with osteoporosis- oral BP users	The study suggests high adherence rates to BP therapy amongst Singaporean patients (mean MPR was 78,9% +/− 27,5% and 69% of the patients were persistent with therapy at 1 year).
Cottéet al. [6]	Retrospective observational study	2,990 women taking-weekly(alendronate or risedronate) or monthly ibandronate.	Adherence to a monthly BP treatment regimen is higher than that to weekly regimens. Patients treated with a monthly regimen were 37% less likely to be non-persistent and were more compliant, with a 5% higher absolute MPR, than women treated with weekly regimens.
Curtis et al. [26]	Randomized controlled study	3,169 women with low bone mass taking placebo	The study found small but significant differences in the change in hip bone mineral density between women with high compliance versus low compliance with placebo.
Briesacher et al. [14]	Cohort study	1,835 individuals who switched to once-monthly BPs	The once-monthly switch was associated with less adherence failure (4% fewer patients per month pre-switch vs. 1% fewer patients per month post-switch; but the impact on fracture risk was uncertain.
Muratore et al. [3]	Randomized controlled trial	60 women with postmenopausal osteoporosis – randomized to two groups: group A: Clodronate (CLD) every month for 12 months, and group B: CLD every 2 weeks for 12 months	A significant increase of BMD in both groups and in both skeletal sites was observed at 12 months versus baseline. No difference was observed between groups. The “twice-a-month” regimen with 200 mg IM CLD may well promote an improved adherence with the same clinical efficacy and safety profile.
Patrick et al. [4]	Cohort study	19,987 patients >65 years old taking BP	The fractures occurred at a rate of 43 to 1,000 people/year, showing an inverse relationship between drug adherence and fracture rate for all measures of adherence and fracture types, excluding distal forearm fractures
Dugard et al. [16]	Cohort study	254 women with osteoporosis	38% patients failed to start treatment, associated with higher BMD Z score and residence in a nursing/residential home. Persistence was associated with a lower comorbidity index and compliance with a lower BMD Z score and fall before starting treatment.
Curtis et al. [7]	Retrospective observational study	101,038 new patients taking BP; 38205 on one or more concomitant therapies	At 1 year, the proportion of persons with high BP compliance (MPR 80%) was 44%. The statin MPR variable was the most significant predictor of 1-year BP compliance, followed by age and prior receipt of BMD testing.
Sheehy et al. [17]	Cohort study	32,804 patients with osteoporosis taking BP oral (weekly alendronate or risedronate)	In the primary prevention cohort, the risk of osteoporotic fractures in the year following BP therapy initiation was reduced by 49% for compliant versus non-compliant patients. In the secondary prevention cohort, the risk of subsequent osteoporotic fracture was reduced by 57% for compliant patients versus non-compliant patients.
Sheehy et al. [8]	Cohort study	32,804 patients taking weekly risedronate or weekly alendronate(brand or generic)	Patients initiated on weekly oral generic alendronate showed a statistically significant lower persistence to BP therapy compared to patients initiated on weekly oral branded risedronate and weekly oral branded alendronate.
Berecki-Gisolf et al. [19]	Cohort study	788 elderly women after osteoporotic fracture- BP users	Adherence to BP treatment by older Australian women with estabilished osteoporosis was poor; within 6 months of starting, half the women stopped their treatment. Adherence failure was more likely among smokers and women taking acid-related medication and less likely among women reporting high levels of physical activity.
Ideguchi et al. [21]	Cohort study	146 patients with osteoporosis - BP users that switched for a second drug	Patients who switched BPs had high rates of persistence of therapy. Those who stopped their first BP because of adverse effects were at risk of discontinuing the second drug for the same reason.

The studies are mostly observational (20 studies), Americans and Europeans, and predominantly involve women over 45 years receiving oral bisphosphonates.

## Discussion

Among studies found, Seven [1,7,11-13,16,19] discussed specifically BPs adherence. 2 studies [1,12] found good adherence to this therapy. In Kuzmanova and colleagues [1], in an experimental study that assessed adherence to the use of ibandronate (monthly) and alendronate, found a high persistence to these BPs in 24 months with MPR of 0.97. The persistence rate was 86.8% at 1 year and 58.94% in 2 years and discontinuation of treatment had rarely been associated with side effects or lack of benefits of medication. Similarly, a Chinese study conducted in patients in Singapore [12] showed high levels of adherence to oral BPs (MPR mean was 78.9% ± 27.5% and 69% of the patients was persistent for the 1 year of therapy). The other five articles, however, did not show similar results. In Curtis and colleagues [7], a study with large number of patients who had started treatment with BPs and recently used other concomitant medications for chronic diseases, the proportion of patients with high compliance (MPR 80%) was only 44% at 1 year, and MPR of statins has been associated with the compliance of BPs. Burden and colleagues [11] have also showed inadequate adherence to BPs (alendronate, risedronate and clodronate): persistence with therapy dropped from 63% at 1 year to 46% in 2 years and 12% in 9 years and most patients discontinued the medication for a time interval for more than once. Similarly, a study [13] showed that oral BPs rate of persistence after 1 and 2 years of 27.9% and 12.9%, respectively, and Berecki-Gisolf [19] and colleagues showed low adherence in Australian women (within 6 months of initiation of therapy, half of the women had stopped treatment) and this was more frequent in women who were smokers and those taking antacids, unlike women who performed regular physical activity. Regarding the failure of treatment initiation, Dugard [16] and colleagues showed that 38% of patients failed to initiate treatment and this was associated with a Z score higher on bone densitometry and residence in “nursing/residential home.”

In 2 studies [20,27], it was examined the association of bone turnover markers with adherence to BPs, with different results. In Roux and colleagues [27], a French multicenter trial that monitored bone turnover markers in patients using ibandronate monthly did not find association of these markers with the persistence use of this medication. In another study [20], it was highlighted that the use of alendronate reduces urinary excretion of N-telopeptide (NTx) and that this reduction is related to compliance.

In studies of Sheehy [8] and Ström [9], they evaluated the use of generic BPs compared to brand, with similar results. In Sheehy and colleagues [8], patients starting generic alendronate weekly had a lower persistence compared to patients taking risedronate or branded alendronate weekly, despite the persistence in general still being inadequate. In the second study [9], the switch of alendronate branded for generic showed reduced persistence.

Several studies have evaluated the association of adherence to some specific factors [2,5,15,18,21,22,24-26]. In Montori [25] and colleagues, using a prevention and treatment osteoporosis guide by patients taking BPs had no impact on adherence after 6 months, but another experimental study [24] evaluating patients taking alendronate or risedronate showed that the group which received counseling treatment had a better adherence. In Devold [18] and colleagues, study conducted with patients taking alendronate, factors associated with adherence were advanced age and high income; in men an average educational level had the greatest impact. In the study of Lai [22] and colleagues, Australian patients who had suffered hip fractures were evaluated, 19.2% of them come from rural areas, and observed that before the fracture less rural patients used BPs (7.7% versus 13.3%) and that after fracture these patients also had lower compliance in relation to the urban group (44% versus 52.4%).

In 3 trials [2,5,15], the treatment satisfaction and the influence of the physician on adherence to medicines were evaluated. A study [2] evaluating physicians and patients of 5 medical centers showed that patients taking alendronate accepted better their illness and treatment when physicians had obtained high scores on a questionnaire that assesses social competence (Social Competence Questionnaire), especially with regard to emotional contact. Barret-Connor [5] and colleagues found an association of poor adherence with patient lower satisfaction with treatment (patients not satisfied had 22 to 67% of increased risk of change of medication or discontinuation of treatment; another study [15] showed that after fracture, patients receiving home clinical visits had more adherence to therapy.

The switch of BPs is common in clinical practice; Ideguchi and colleagues [21] found that patients who switched medication had higher rates of persistence to BPs, but those who stopped at the first BP due to side effects had risk to discontinue the second BP for the same reason.

Another interesting experimental study [26] evaluated in one arm, the use of placebo in patients with low bone density, and found differences in hip BMD between groups of high compliance and low compliance, but more studies are needed to confirm this finding.

In 2 studies [4,17], adherence in patients with osteoporotic fracture or the impact of adherence in preventing fractures were evaluated with similar results. In Patrick and colleagues [4], a study conducted with large number of elderly patients, there was an inverse relationship between adherence and fracture rate (except limbs): persistent increase was associated with a 22% reduction in all fractures, 23% reduction in hip fractures and 26% reduction in the rate of vertebral fractures. Similarly, in another study [17] conducted with patients taking alendronate or risedronate, the risk of osteoporotic fractures in the first year of therapy with BPs was reduced by 49% for compliant versus non-compliant patients. Also in this study, the group that had already suffered fractures, the risk of new fractures was reduced by 57%.

BPs may be used in different doses. The last 5 studies [3,6,10,14,23] included, assessed the relationship between adherence and dosage of these drugs. In three studies [6,10,14] the results were similar. In Cotte and colleagues [6], patients taking alendronate or risedronate weekly and taking ibandronate monthly were evaluated: monthly dosage achieved greater adherence when compared to weekly dosage (monthly dose patients were 37% less likely to be non-persistent and were more compliant (MPR 5% higher). Devine and colleagues [10], in a study conducted in the U.S. Military Health System, also showed better compliance in patients with monthly dose of BPs, compared to weekly dosage (patients percentage of high MPR 45.7% within the monthly dosage group versus 42.2% in the group of weekly dosage). Briesacher and coleagues [14] evaluated patients who switched BPs showed that those who switched to monthly dosage had less non-adherence (4% fewer patients per month pre-switch versus 1% fewer patients per month post-switch).

The clodronate was evaluated in an experimental study [3], in 2 different doses: 100 mg intra-muscular (IM) monthly for 12 months or 200 mg IM every 2 weeks for 12 months: in relation to the increase in BMD there were no differences between groups, but the fortnightly dose showed greater adherence of patients. Study [23] comparing patients taking ibandronate and zolendronate, showed that the latter group had more adherence.

This review is relevant to health sciences area, since it provides also important information for the growth and development area [28,29]. Therefore, it helps to understand a part of the mechanisms involving this drug.

## Conclusion

Adherence to BPs, in most American and European studies, is still unsatisfactory. However, a Chinese study conducted in Singapore and other European study conducted in Bulgaria showed good adherence to treatment, with high rates of persistence. A monthly dosage is associated with better adherence compared to weekly dosage, despite the methodological limitations of the studies. Therefore, there is a need for more experimental studies, given that the studies are mostly observational (predominantly cohort), to offer further information in relation to these drugs.

## Competing interest

We declare no conflict of interest.

## Author’s contribution

All authors participated in the acquisition of data and revision of the manuscript. IAL, TMAS, JAP and ANA interpreted the data and drafted the manuscript. EPV, VEV, LCA and FA determined the design and drafted the manuscript. All authors read and gave final approval for the version submitted for publication.
